# How robust are the natural history parameters used in chlamydia transmission dynamic models? A systematic review

**DOI:** 10.1186/1742-4682-11-8

**Published:** 2014-01-30

**Authors:** Bethan Davies, Sarah-Jane Anderson, Katy ME Turner, Helen Ward

**Affiliations:** 1 School of Public Health, Imperial College London, St Mary’s Campus, Praed Street, London W1 2PG, UK; 2 School of Social and Community Medicine & School of Clinical Veterinary Science, University of Bristol, Bristol BS8 2PS, UK

**Keywords:** Chlamydia trachomatis, Mathematical modelling, Systematic review, Natural history, Screening

## Abstract

Transmission dynamic models linked to economic analyses often form part of the decision making process when introducing new chlamydia screening interventions. Outputs from these transmission dynamic models can vary depending on the values of the parameters used to describe the infection. Therefore these values can have an important influence on policy and resource allocation. The risk of progression from infection to pelvic inflammatory disease has been extensively studied but the parameters which govern the transmission dynamics are frequently neglected. We conducted a systematic review of transmission dynamic models linked to economic analyses of chlamydia screening interventions to critically assess the source and variability of the proportion of infections that are asymptomatic, the duration of infection and the transmission probability. We identified nine relevant studies in Pubmed, Embase and the Cochrane database. We found that there is a wide variation in their natural history parameters, including an absolute difference in the proportion of asymptomatic infections of 25% in women and 75% in men, a six-fold difference in the duration of asymptomatic infection and a four-fold difference in the per act transmission probability. We consider that much of this variation can be explained by a lack of consensus in the literature. We found that a significant proportion of parameter values were referenced back to the early chlamydia literature, before the introduction of nucleic acid modes of diagnosis and the widespread testing of asymptomatic individuals. In conclusion, authors should use high quality contemporary evidence to inform their parameter values, clearly document their assumptions and make appropriate use of sensitivity analysis. This will help to make models more transparent and increase their utility to policy makers.

## Background

Chlamydia screening programmes have been implemented with the aim of reducing the incidence and prevalence of infection and its complications [1,2]. As there are few randomised controlled trials (RCTs) looking at the impact of screening on these outcomes, [3] transmission dynamic models are used to provide insight into their likely impact [4,5]. Estimates from these models, such as the number of infections over time, are frequently used in economic models (often in the form of a decision tree) to assess the cost-effectiveness of hypothetical chlamydia screening programmes. Therefore dynamic economic models are an important source of evidence for policy makers.

Transmission dynamic models capture not only the direct effects of screening (i.e. the number of individuals screened, diagnosed and treated) but also the indirect effect of reduced onward transmission [6]. A key concept in infectious disease dynamics is the ‘basic reproduction number’ (R_0_); the average number of new infections arising from an infected individual in a wholly susceptible population [7]. It is determined by both population level factors and biological factors specific to the organism and is the product of the probability of transmission between an infected person and an uninfected person (β), the contact rate between infected and susceptible people (c) and the duration of infection (D).

Despite this fundamental role in infection transmission dynamics, there is widely acknowledged uncertainty in the parameters used to calculate R_0_ for chlamydia [8-10]. This means that the predicted impact of a chlamydia screening intervention can be altered under different but similarly plausible assumptions about for example, the untreated duration of infection or the proportion of infections that are asymptomatic [10,11]. To improve the interpretation of the results from a model it is important to consider how the assumptions used in the study compare to the full range of plausible input values.

In this study we focus on transmission dynamic models embedded within economic analyses. We will consider the representation of the biological characteristics of chlamydia that are central to modelling its transmission in a population, and that ultimately influence the predicted success of interventions. Specifically, we aim to critically assess the source and variability of the proportion of infections that are asymptomatic, the duration of infection and the transmission probability to increase model transparency and improve understanding of the current level of knowledge on the biological features determining the transmission of chlamydia.

## Methods

We searched Pubmed, Embase and the Cochrane database between 01/01/2004 and 29/05/2013 using a search strategy based on a published systematic review of economic evaluations of chlamydia screening [12]. For full details of the search methodology see additional information (Additional file 1). To be included in this review, studies must contain a transmission dynamic model linked to an economic model that considers women in a general population setting, any chlamydia screening intervention and at least one adverse reproductive outcome in women. The search was limited to models with an economic component as they are formulated to directly inform the policy making process (such studies must include at least one reproductive outcome in order to estimate the potential health benefits from screening). Non-English language and non-human studies, abstracts, letters and editorials were excluded.

References were imported to an Endnote library where duplicates were identified based on author, title and year of publication followed by a manual search for additional duplicates. One author (BD) screened the titles, abstracts and full manuscripts, where necessary, to identify studies for inclusion. Two authors (SA and BD) reviewed the reference lists of included articles for relevant studies and extracted information about study design, setting, natural history parameter values and parameter source. We limited the natural history parameters to those which influence the transmission dynamic component of the model. Other parameters, including those which determine the likelihood of later complications, have been reviewed elsewhere and were beyond the scope of this review [13]. For each study included in the review, we obtained the references for the natural history parameters and the references cited within these studies (if they were not original studies) until the underlying reference was identified.

We present a descriptive analysis of the natural history parameters and a critical review of the quality and appropriateness of the cited sources. For the proportion of infections which are asymptomatic in women, we constructed a flowchart depicting the pathway of references used to inform the assumption in each of the studies included in this review. Within this figure, we classified the studies as either "primary" studies if they generated new data or "review or modelling" studies if they were based on existing data. We also identified the primary studies that had a suitable design for informing this parameter (defined as a study looking at a population of women not selected on the basis of symptoms who were tested for chlamydia) and presented a parameter value.

## Results

**Figure 1:**
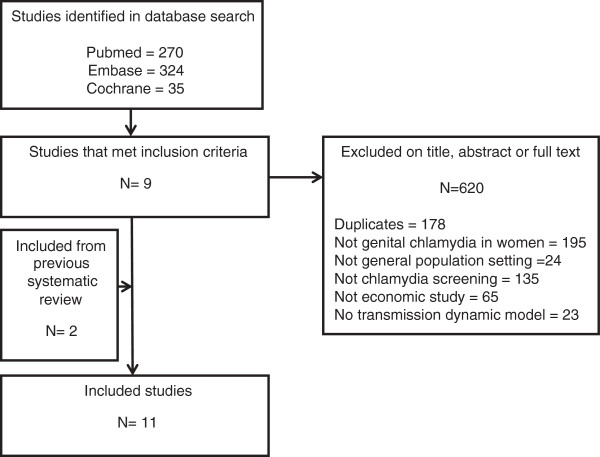
Identification of eligible studies.

The initial search identified 629 papers of which 9 met the inclusion criteria [5,6,14-20]. We also included two studies from the systematic review we based our search strategy on that met the inclusion criteria for this study [21,22] (Figure 1). These eleven studies included two sets of publications from research groups that use the same dynamic model and parameter values [6,16,17,22]. To avoid over-representing the methods of these authors we have combined the parameter information from each set of papers and we reference the earliest publication when referring to the set [17,22]. Therefore we present our analysis of nine studies with independent parameter estimates [5,14,15,17-22].

**Figure 2:**
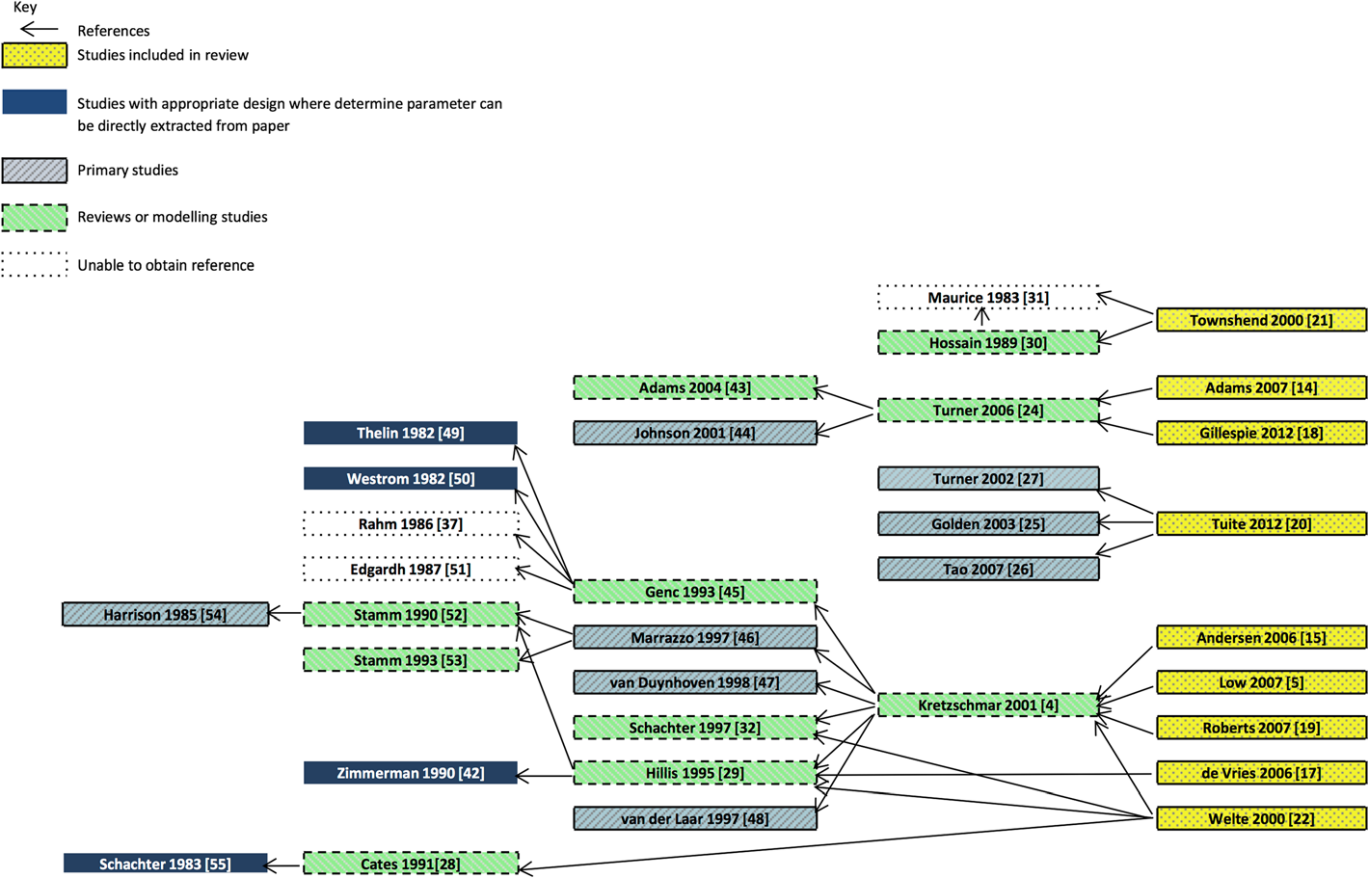
Illustration of reference pathway for "proportion of infections that are asymptomatic in women".

All the studies included in this review consider the cost-effectiveness of hypothetical chlamydia screening programmes or modifications to existing control strategies in developed countries (Table 1). Six of these nine studies [5,14,15,18,19,22] use an individual based transmission dynamic model and the remaining three [17,20,21] use a compartmental model. Two of the nine studies [17,21] developed a novel transmission dynamic model and 7 studies [5,14,15,18-20,22] are based on one of three previously published transmission dynamic models, Fisman et al. [20,23]; Kretzschmar et al. [4,5,15,19,22] and Turner et al. [14,18,24]. The values of key parameters used in the included studies are given in Table 1. Figure 2 provides an illustration of the reference pathway for the proportion of infections that are asymptomatic in women [4,5,14,15,17-22,24-47]; the reference pathway for all three parameters, in men and women, is provided in the additional information (Additional file 2).

**Table 1:** Description of included studies

**Study**	**Setting**	**TD model structure and source**	**Screening uptake source**	**Baseline chlamydia prevalence source**	**Proportion asymptomatic**	**Duration of infection**		**Risk of transmission (baseline)**
						**No symptoms**	**Symptoms**	
**Adams et al. 2007 **[14]	Comparison of screening strategies, UK	Individual based, Turner et al. [24]	Studies of opportunistic screening, England	Systematic review and UK survey data	95.5% women; 100% men	180 days not seeking treatment	30 days seeking treatment	0.0375 per act
**Andersen et al. 2006 **[15]	Home sampling screening with partner notification, Denmark	Individual based, Kretzschmar et al. [4,48]	RCT of home sampling, Aarhus	Danish surveillance system and observational study in Aarhus	70% women; 50% men	370 days in women; 200 days in men	40 days in women; 33 days in men	0.11 per act
**de Vries et al. 2006 **[17]	One off screening, the Netherlands	Compartmental, original model	Pilot of one off screening, the Netherlands	Pilot of one off screening, the Netherlands	70% women; 50% men	1 year	1 month	0.68 assume per partnership
**de Vries et al. 2008 **[16]	Repeat systematic screening, the Netherlands	As above	As above	As above	As above	As above	As above	As above
**Gillespie et al. 2012 **[18]	Opportunistic screening, Ireland	Individual based, Turner et al. [24]	Pilot of opportunistic screening, Ireland	UK data	95.5% women; 100% men	180 days	30 days	0.0375 per act
**Low et al. 2007 **[5]	Active screening, UK	Individual based, Kretzschmar et al. [4,48]	ClaSS cross sectional study of screening uptake	ClaSS project	70% women; 25% men	200 days	40 days in women; 33 days in men	0.122 per act female to male; 0.154 per act male to female
**Roberts et al. 2007 **[19]	Register based screening, England	Individual based, Kretzschmar et al. and Low et al. [4,5,48]	ClaSS cross sectional study of screening uptake	ClaSS project	70% women; 25% men	200 days	40 days in women; 33 days in men	0.061 per day female to male;
								0.077 per day male to female
**Townshend and Turner 2000 **[21]	Three different screening strategies, UK	Compartmental, original model	Not presented	Sample of women presenting for cervical smear, UK	75% women; 50% men	2-3 years	Not presented	Not presented
**Tuite et al. 2012 **[20]	Screening, Canada	Compartmental, Fisman et al. [23]	Testing patterns from Ontario Public Health Laboratory	Annual notifiable disease data, Canada	90% women; 92% men	1 year untreated	Not presented	Present per partnership transmission probability* partner change rate
**Welte et al. 2000 **[22]	GP based opportunistic screening, Netherlands	Individual based, Kretzschmar et al. [4,48]	GP pilot study, Amsterdam	GP pilot study, Amsterdam	70% women; 50% men	Not presented	Not presented	0.10 per act
**Welte et al. 2005 **[6]	As above	As above	As above	As above	Not stated, assume as above	370 days in women; 200 days in men	40 days in women; 33 days in men	0.11 per act

All nine studies assume a sex difference in the proportion of the population who develop symptoms following infection. In three of the nine studies, men have a higher proportion of infections that are asymptomatic (92-100% compared to 90–95.5% in women) [14,18,20]. Two of this group of three studies [14,18] share a common source for this parameter, the modelling study by Turner et al., [24] where the proportion of infections that are asymptomatic is obtained through model fitting (Figure 2). The other study in this group of three [20] references a variety of studies [25-27]. The remaining six studies assume that between 70-75% of infections in women are asymptomatic [5,15,17,19,21,22]. Four of these six assume that 50% of infections in men are asymptomatic [15,17,21,22] and two assume that 25% of infections in men are asymptomatic [5,19]. The modelling study by Kretzschmar et al. is referenced as the sole source of this parameter in three of the six studies [4,5,15,19] while the remaining three studies [17,21,22] reference a variety of studies [28-32,49].

For the duration of infection, seven of the nine studies define it for symptomatic or asymptomatic infection [5,15,17-19,21,22] and two define it for treated or untreated infection [14,20]. For the purposes of this comparison, when discussing duration of infection we have included estimates of the duration in treated individuals in the ‘symptomatic’ category and estimates of the duration in untreated individuals in the ‘asymptomatic’ category, although we recognise that this is an assumption. Five of the nine studies assume no sex difference in the duration of infection, [14,17,18,20,21] two assume a sex difference in the duration of symptomatic infection [5,19] and two assume a sex difference in the duration of symptomatic and asymptomatic infections [15,22]. Where a sex difference is assumed, infection is always stated to last longer in women. In eight of the nine studies, asymptomatic infection is estimated to last between 180–370 days in women and 180–200 days in men [5,14,15,17-20,22]. In the remaining study, the duration of asymptomatic infection is stated to be around 2–3 years [21]. The duration of symptomatic infection ranges between 30–40 days in women and 30–33 days in men in the seven studies where this parameter is presented [5,14,15,17-19,22].

Adams and Gillespie [14,18] assume the shortest duration of symptomatic and asymptomatic infection and are based on the model by Turner et al. where the parameters were obtained from reviews by Golden et al. and Korenromp et al. [24,50,51]. Andersen, Low, Roberts and Welte [5,15,19,22] base their durations on the model by Kretzschmar et al. [4] who referenced prospective cohorts by Buhaug et al. [52] and Rahm et al. [33] for the duration of asymptomatic infection in women while the remaining durations (symptomatic infection in women; asymptomatic infection in men; symptomatic infection in men) were taken from a technical report by van de Laar et al. and based on studies of gonorrhoea as there is no equivalent data for chlamydia [53]. De Vries and Tuite use one year as the duration of asymptomatic infection in women [17,20] based on a cost-effectiveness study by Buhaug et al. and a review by Geisler et al. [9,54]. One study does not reference this parameter [21].

Five of the nine studies in this review use a per act transmission probability of between 0.0375 and 0.154 [5,14,15,18,22]. Of these five studies, the two [14,18] that use the lowest value of 0.0375 cited the modelling study by Turner et al. who obtained their value by fitting model prevalence to prevalence data [24]. The other three of these five studies [5,15,22] cite the modelling study by Kretzschmar et al. who estimated that the upper bound for the per act transmission probability is 0.108, (calculated from a Sexually Transmitted Infection (STI) clinic study of concordance in partnerships by Quinn et al.) [4,55,56]. Of the four remaining studies, one [17] uses data from the same study by Quinn et al. and a per partnership transmission probability of 0.68 [56], one uses a per day transmission probability based on the model of Kretzschmar et al. [4,19] and two do not explicitly state the value used for this parameter [20,21]. Of the seven studies that present the risk of transmission, two assume a sex difference with a higher risk from male to female [5,19].

The majority of the studies (8 out of 9) perform a sensitivity analysis [5,14,15,18-22]. However these often focus on intervention related parameters (including the probability of accepting a screen [5,14,18-22], level of partner notification [15,22], efficacy of treatment [14,18,20,21]) and the risk of complications [5,14,15,19-22]. The natural history parameters considered in this review are often estimated through model fitting or calibration to prevalence or incidence data and the final values used in the models are sometimes not stated.

The quality of referencing for the parameter values was variable. Multiple papers were often cited for each parameter (Figure 2 and Additional file 2) and where it was possible to identify the source reference, a high proportion of parameter values were referenced back to the earlier transmission dynamic models [4,23,24]. Some references were from clinical studies conducted before nucleic acid amplification tests (NAATs) and widespread testing of asymptomatic individuals (e.g. Rahm et al. 1986 [33] (duration of infection) and Zimmermann et al. 1990 [34] (proportion asymptomatic)). Also, parameter values were often modified from the figure given in the referenced primary study, with varying levels of detail about the rationale behind this change. For the proportion of infections that were asymptomatic in women, we were able to access 22 of the studies that make up the reference pathways of the nine studies in this review. Of these 22 studies, we consider that 12 were primary studies and 4 had an appropriate design to inform estimates of this parameter.

## Discussion

### Main findings

We found wide variation in the natural history parameters used in transmission dynamic models of chlamydia linked to economic analyses. There was an absolute difference in the proportion of asymptomatic infections of 25% in women and 75% in men, a six-fold difference in the duration of asymptomatic infection and a four-fold difference in the per act transmission probability. We conclude that much of this variation can be explained by a lack of evidence or consensus in the literature as multiple sources were often referenced for each parameter. Additionally, there was often little discussion of how the final parameter value was chosen. This may be due to word count restrictions but a description of this critical process would allow readers to better critique the appropriateness of the parameter values used and the potential generalizability of the findings.

### Strengths and limitations of study

We performed a systematic search of the literature. We limited our search to English language papers but we used broad search terms (based on an earlier systematic review) to try and capture relevant studies. We restricted our analysis to studies with transmission dynamic models as this is the gold standard for the cost-effectiveness assessments that are predominantly used by policy makers [12,57]. Our analysis was limited by the availability of historic references for parameter values and by the reporting and referencing of parameter values in the source publications.

### Role of model structure and model fitting

Model fitting is commonly used with transmission dynamic models. This is the process of systematically varying the initial parameter values (e.g. transmission probability) until the output of the model reflects a known parameter (e.g. chlamydia prevalence) in the studied population. However different parameter combinations can sometimes predict the same model output. Model fit can be improved by fitting to more than one measurement (e.g. prevalence and incidence) but when parameters are obtained through fitting, the model output can only ever be as good as the underlying empirical estimates. Furthermore, correlation exists between certain parameters, such as the transmission probability and duration of infection. This introduces an additional complexity as it means that assumptions made about one variable can and will impact on the other parameter. Therefore to fully interpret a model output it is necessary to understand the uncertainties in each parameter, the correlation between parameters and the underlying model structure.

Meta modelling studies have shown how both model uncertainty and parameter uncertainty can lead to disparate conclusions despite plausible baseline prevalence estimates [10,58]. The appropriate use of sensitivity analysis can explore whether the natural history parameters are affecting the cost-effectiveness findings or the potential impact of the intervention. These issues surrounding model structure, fitting and assumptions have been discussed extensively and there are guidelines available detailing best practice for modelling studies [57].

### Strengths and limitations of parameter values

The majority of parameters were well referenced and appropriately sourced and we assume that many of those without a reference were obtained through model fitting. However we found several examples of authors citing several different sources or using parameter values that differed from the quoted source without an adequate discussion of the rationale behind the change. Few authors fully discussed the implications of using parameter values from other settings despite a body of evidence describing how the epidemiology of chlamydia and its complications varies between regions and has changed over time.

The overarching limitation of parameter values used in transmission dynamic models is that they are obtained from primary studies that are not always able to measure the underlying process that will be modelled. For example, we will never be able to measure the true duration of a chlamydia infection in an observational study, as it is the timing of diagnosis rather than the timing of infection that is measured. The design and conduct of the primary study may also affect how well its estimates reflect the true situation. For example the ability of a result of a diagnostic test to measure the true situation is limited by its sensitivity and specificity. And estimates from primary studies may differ between settings and over time, for example due to the introduction of more sensitive tests or a change in the risk profile of the population being tested.

Therefore there will always be uncertainty around how well the estimates from primary studies represent the true situation being modelled. In addition, these estimates (with an uncertain relationship to the true parameter value) are then used in models which by definition are a simplification of the real situation and may not be able to reflect the breadth of heterogeneity in real life. With these limitations in mind, we will go on to discuss the specific strengths and limitations of the three natural history parameters considered in this review.

There was general agreement that asymptomatic infection is more common in women than men (Table 2). This may reflect the true biology of chlamydia. However if infection lasts longer in women (e.g. faster clearance from the male urethra) there is more opportunity to detect prevalent, and arguably asymptomatic, infections in women. This means that if the true likelihood of having symptoms during an infection is equal between the sexes, a longer duration of infection in women may act to increase the number of infections that are asymptomatic at detection as a proportion of all detected infections in women.

**Table 2:** Summary of chlamydia natural history parameters used in included studies and suggested evidence based parameter values for future transmission dynamic models

**Parameter**	**Range used in included papers**	**Suggested value**
**Proportion asymptomatic**	Male: 25% - 100%	To be based on observed treatment seeking rates in the modelled population [24]
	Female: 70% - 95.5%	
**Duration of infection**		
**a) Symptomatic**	Male: 30 – 33 days	30 - 33 days (consensus)
	Female: 30 – 40 days	
**b) Asymptomatic**	Male: 180 days – 3 years	497 days [59]
	Female: 180 days – 3 years	
**Risk of transmission per act**	0.0375 – 0.154 male - female	0.095 (IQR 0.06 – 0.167) [60]
	0.0375 – 0.122 female - male	

The commonest figures for the proportion of asymptomatic infections, 70% in women and 50% or under in men, are from a historical contact tracing study which used tests with low sensitivity [34]. Therefore this study may underestimate the true proportion infected. If we are going to continue to use this parameter in models there is a need to obtain a contemporary estimate from cases diagnosed using highly sensitive NAATs.

However there is an alternative approach. It has been shown that using a population specific measure of the proportion of individuals who seek treatment, rather than the proportion of infections that are asymptomatic, can provide a better fit to observed chlamydia prevalence [24]. Using observed data on treatment seeking behaviour bypasses the need to make assumptions about the underlying proportion of infections that are symptomatic and ensures that the measure is specific to the population of interest. This is important as the proportion of infections that are symptomatic may not be generalizable as symptom recognition may vary between populations [51].

Systematic reviews have failed to find consensus in the estimated duration of untreated chlamydia infection [9,50,51] which may explain why a range of values are used in the studies included in this review (from 180 days to 2–3 years). Most studies assume a shorter duration of infection in men despite little evidence for such a difference [50]. Many authors have discussed the enormous challenges to accurately measuring the duration of untreated infection, including the unknown time from infection to diagnosis, the possibility of reinfection (with same or different strain) and length-time bias from screening [9,50,59]. Price et al. evaluated this variation in duration of infection using a mathematical model and predicted that there are several classes of infection that clear at different rates [59]. They estimate that the overall mean duration of untreated infection is 1.36 years (497 days) (95% CI 413–595 days). This is longer than the previously assumed 1 year median duration reported by a systematic review [9] but similar to the estimate of 433 days (95% CI 420–447) from a modelling study [11]. If these modelling studies are considered to provide more reliable contemporary evidence than historical clinic-based studies alone, then the majority of the studies in this review have truncated the true duration of untreated infection. This may have led to an underestimation of the predicted impact of the screening intervention which in turn may lead to conservative policy decisions.

We found two studies that assumed a sex difference in the probability of transmission. It is likely that this assumption is based on analogy to gonorrhoea, [48] although it has been stated that there is no evidence to suggest that the risk of transmission in chlamydia differs by direction [56]. The majority of studies in this review based the transmission probability on a historic cross-sectional study of STI clinic patients which found that 68% of partners of women diagnosed with chlamydia were also infected [56]. This figure is incorrectly taken to indicate a transmission probability of 0.68. Other authors have discussed the problems with inferring transmission probability from concordance [56,60].

A per act transmission probability is more difficult to measure than the per partnership probability, but it allows reinfection within a partnership to be modelled (which is important if repeat infections have a different risk of complications). Katz et al. used data from a contact tracing programme to estimate that the per act transmission probability was 0.395 from men to women and 0.323 from women to men, [55] although they highlight a number of limitations, including assumptions about the distribution of the transmission probability per act with duration of partnership and uncertainty in the number of sex acts within a partnership [8,55,60]. Althaus et al. used Quinn’s raw data in a pair model to estimate that the "true" partnership transmission probability, based on two partnerships in the last 12 months, is 0.55 (IQR 0.49 - 0.63) and the "true" per act transmission probability is 0.10 (IQR 0.06 to 0.17), although the limitations with the raw data remain [60].

## Conclusion

As model predictions are increasingly used to inform public health policy, there is an urgent need for further empirical research to reduce parameter uncertainty. Contemporary estimates could be obtained or improved by undertaking research including surveys to estimate treatment seeking behaviour, mathematical modelling to improve estimates of the duration of infection and concordance studies within screening programmes (in the absence of expedited partner therapy) to estimate the risk of transmission. In the meantime, authors should use the highest quality contemporary evidence to inform their parameter values, clearly document their assumptions and make appropriate use of sensitivity analysis. This will help to make models more transparent and increase their utility to policy makers.

## Abbreviations

CI: Confidence interval; IQR: Inter quartile range; NAAT: Nucleic acid amplification test; RCT: Randomised controlled trial; STI: Sexually transmitted infection.

## Competing interests

BD and HW are currently working on an ECDC project to review Chlamydia Control Guidance. SA and KT declare that they have no competing interests.

## Authors’ contributions

BD was responsible for the study concept. BD and SA conducted the initial literature search, extracted information from the included studies and co-wrote the first draft. All authors contributed to the interpretation of the data and critical review of the manuscript. All authors read and approved the final version for publication.

## Authors’ information

BD, MB BChir MA MPH MFPH is a Clinical Research Fellow; SA, BA Hons MSc is a Research Assistant; KT, PhD is a Senior Lecturer; HW, MB ChB MSc PhD FRCP FFPH is Professor of Public Health.

Bethan Davies and Sarah-Jane Anderson are joint-first authors.

## Supplementary Material

Additional file 1 **Systematic search methodology.** The file contains a detailed description of the systematic search of the literature performed for this review. Click here for file

Additional file 2 **Summary of key references used to support parameter values.** The file contains a brief description of the references cited as parameter sources by the studies included in the review. Click here for file
