# Supplementary material for: How robust are the natural history parameters used in chlamydia transmission dynamic models? A systematic review
Source: Theor Biol Med Model. 2014 Jan 30;11:8. doi: 10.1186/1742-4682-11-8 (PMC3922653; doi:10.1186/1742-4682-11-8)
Supplement: Additional file 1 — Systematic search methodology. The file contains a detailed description of the systematic search of the literature performed for this review. [file 1742-4682-11-8-S1.docx]

**Additional file 1**

Systematic search methodology

**Objective**

To systematically identify published transmission dynamic models linked to economic evaluations of chlamydia screening interventions and to extract the chlamydia natural history parameters and their sources.

**Method**

The inclusion criteria for this study are defined as:

1. Population: to include women in a general population setting
2. Intervention: any chlamydia screening intervention
3. Outcome: Pelvic Inflammatory Disease (PID), Chronic Pelvic Pain, Ectopic Pregnancy (EP) or Infertility
4. Study design: a transmission dynamic model of chlamydia linked to an economic model
5. English language

The exclusion criteria for this study are defined as:

1. Non-human studies
2. Abstracts, letters and editorials

We based our search strategy on a previously published systematic review of economic evaluations of chlamydia screening. [[1](#_ENREF_1), [2](#_ENREF_2)] The inclusion criteria for this study are broader than those used in this study, therefore we assume that all studies meeting the inclusion criteria for our review published prior to 2004 will have been identified by this study. One author (BD) searched the online databases Pubmed, Embase and the Cochrane Collaboration from 01/01/2004 to 29/05/2013, and extracted papers included in the earlier systematic review that met the inclusion criteria for this study. [[1](#_ENREF_1), [2](#_ENREF_2)]

1. PUBMED – on 29.05.13

| **#1** | "Chlamydia"[All fields] OR "chlamydia infections"[ All fields] OR "Chlamydia trachomatis"[ All fields]) | 23197 |
| --- | --- | --- |
| **#2** | "economics"[MESH] OR "economic evaluation"[ALL FIELDS] OR "cost benefit analysis"[MESH] OR "cost"[ALL FIELDS] OR "cost analysis"[ALL FIELDS] OR "cost effectiveness analysis"[ALL FIELDS] OR "cost utility analysis"[ALL FIELDS] OR "cost minimisation"[ALL FIELDS] OR "quality of life"[MESH] OR "QALY"[MESH]) | 711686 |
| **#3** | #1 AND #2 | 812 |
| **#4** | #3 AND ("2004/01/01"[PDAT] : "2013/05/28"[PDAT]) | 324 |
| **#5** | #4 AND “humans” [MESH] | 296 |
| **#6** | #5 AND ((Clinical Trial[ptyp] OR Journal Article[ptyp] OR Meta-Analysis[ptyp] OR Multicenter Study[ptyp] OR Randomized Controlled Trial[ptyp] OR Review[ptyp] OR systematic[sb] OR Technical Report[ptyp] OR Guideline[ptyp] OR Evaluation Studies[ptyp] OR Comparative Study[ptyp]) | 278 |
| **#7** | #6 AND “English” {lang} | 270 |

1. EMBASE – on 29.05.13

| **#1** | Chlamydia.mp | 28996 |
| --- | --- | --- |
| **#2** | “economics.mp” OR “exp economic evaluation/” OR “exp "cost benefit analysis"/” OR “Exp cost/” OR “exp "cost effectiveness analysis"/” OR “exp "cost utility analysis"/” OR “exp "cost minimization analysis"/” OR “exp "quality of life"/” OR “QALY.mp” | 755587 |
| **#3** | #1 AND #2 | 865 |
| **#4** | #3 AND "human" [Subjects] | 792 |
| **#5** | #4 and 2004:2013.(sa_year). | 413 |
| **#6** | Letter.pt | 823778 |
| **#7** | Abstract.pt | 1035612 |
| **#8** | Editorial.pt | 431834 |
| **#11** | #5 not (#6 or #7 or #8) | 342 |
| **#12** | #11 limited to English language | 324 |

1. COCHRANE – on 21.05.13

| **#1** | chlamydia in title abstract keywords in Economic Evaluations, 2004-2013 | 35 |
| --- | --- | --- |

All references were imported to an Endnote library where duplicates were identified based on author; title and year of publication followed by a manual search for additional duplicates. One author (BD) screened the titles, abstracts and full manuscripts where necessary, to identify eligible studies. Additional studies that met the inclusion criteria for this review were included from the earlier systematic review of economic evaluations [[1](#_ENREF_1)] and two authors (SA and BD) reviewed the reference lists of included studies for further eligible publications.

Two authors (SA and BD) read the included studies and extracted information about the study design and setting and the pre-specified chlamydia natural history parameter values used in the transmission dynamic model together with the range used in sensitivity analyses, the cited source(s) of each parameter and any documented assumptions (Table). It was beyond the scope of this review to consider factors related to the progression of chlamydia to complications and the costs and cost-effectiveness methodology used in the economic evaluation.

Table: Information extracted from included studies

| **General study characteristics** | **Parameters from transmission dynamic model** | **Source of parameters** |
| --- | --- | --- |
| - Setting | - Risk of transmission | - References cited as the source of the three parameters |
| - Intervention | - Proportion asymptomatic |  |
| - Source of transmission dynamic model | - Duration of infection |  |
| - Source of screening uptake estimate |  |  |
| - Source of chlamydia prevalence estimate |  |  |

**References**

1. Roberts, T.E., et al., *Screening for Chlamydia trachomatis: a systematic review of the economic evaluations and modelling.* Sex Transm Infect, 2006. **82**(3): p. 193-200; discussion 201.

2. Low, N., et al., *Epidemiological, social, diagnostic and economic evaluation of population screening for genital chlamydial infection.* Health Technol Assess, 2007. **11**(8): p. iii-iv, ix-xii, 1-165.
