# Supplementary material for: How robust are the natural history parameters used in chlamydia transmission dynamic models? A systematic review
Source: Theor Biol Med Model. 2014 Jan 30;11:8. doi: 10.1186/1742-4682-11-8 (PMC3922653; doi:10.1186/1742-4682-11-8)
Supplement: Additional file 2 — Summary of key references used to support parameter values. The file contains a brief description of the references cited as parameter sources by the studies included in the review. [file 1742-4682-11-8-S2.docx]

**Additional file 2**

Summary of key references used to support parameter values

| Parameter | Reference | Description | Cited by |
| --- | --- | --- | --- |
| Proportion asymptomatic | Kaufman 1974 [[1](#_ENREF_1)] | Design: Review  Limitations: Describes non-gonococcal urethritis (NGU)  Main findings: The proportion of cases of NGU that are attributable to chlamydia and the presence of additional symptoms in men with NGU | Genc 1993 [[2](#_ENREF_2)] cited by Kretzschmar [[3](#_ENREF_3)] cited by [[4-7](#_ENREF_4)] |
|  | Podgore 1982 [[8](#_ENREF_8)] | Design: Cross-sectional study of 97 asymptomatic male U.S. military personnel  Limitations: Study design is not suitable for determining the proportion of infections that are asymptomatic  Main findings: 11.3% (11/97) of men were chlamydia positive | Stamm 1986 cited by [[7](#_ENREF_7)] and Hillis 1995 cited by [[7](#_ENREF_7), [9](#_ENREF_9)] |
|  | Thelin 1982 [[10](#_ENREF_10)] | Design: Cross-sectional study of contact tracing  Limitations: Study population are partners of chlamydia infected people  Main findings: 127 female partners of 155 men infected with chlamydia; 66.1% (84/127) of women were infected; overall 51.2% (65/127) of women who were “harbouring chlamydiae” were asymptomatic | Genc 1993 [[2](#_ENREF_2)] cited by Kretzschmar 2001 [[3](#_ENREF_3)] cited by [[4-7](#_ENREF_4)] |
|  | Westrom 1982 [[11](#_ENREF_11)] | Design: Cohort study of women of reproductive age  Limitations: Women eligible for screening were presenting for abortion or contraceptive services; women in the non-screening group may have been asymptomatic  Main findings: 56.0% (7671/13,697) of the population were tested for chlamydia during the study period; 6.2% (622/9,995) of culture samples were defined as screening samples; 3.0% (30/1,013) of the women with a positive chlamydia culture had a screening test rather than a test due to symptoms/signs or an infected partner. The ratio of symptomatic to asymptomatic women by age is presented, 2:1 for 15-19 year olds | Genc 1993 [[2](#_ENREF_2)] cited by Kretzschmar et al 2001 [[3](#_ENREF_3)] cited by [[4-7](#_ENREF_4)] |
|  | Maurice 1983 [[12](#_ENREF_12)] | Full text article not available | [[13](#_ENREF_13)] |
|  | Schachter 1983 [[14](#_ENREF_14)] | Design: Cross-sectional study of 3,020 women attending family planning clinic  Limitations: Chlamydia diagnosed using culture of endocervical specimens  Main findings: Overall chlamydia prevalence was 9.8%; clinical information was collected from 1,230 women; “70% of chlamydial infections were clinically inapparent” in women | Cates 1991 [[15](#_ENREF_15)] cited by [[7](#_ENREF_7)] |
|  | Adger 1984 [[16](#_ENREF_16)] | Design : Cross-sectional study of 50 adolescent males attending an STI clinic  Limitations: Primary outcome is performance of first-catch urine  Main findings: 78.0% (39/50) of men were asymptomatic; 28.2% (11/39) of the asymptomatic men were culture positive for chlamydia | Randolph 1990 [[17](#_ENREF_17)] cited by Genc 1993 [[2](#_ENREF_2)] cited by Kretzschmar 2001 [[3](#_ENREF_3)] cited by [[4-7](#_ENREF_4)] |
|  | Stamm 1984 [[18](#_ENREF_18)] | Design: Cross-sectional study of chlamydia prevalence in 596 men at an STI clinic  Limitations:  Main findings: 73% of heterosexual men and 35% of homosexual men with positive chlamydia cultures had symptoms | Stamm 1986 cited by [[7](#_ENREF_7)], Randolph 1990 [[17](#_ENREF_17)] cited by Genc 1993 [[2](#_ENREF_2)] cited by Kretzschmar 2001 [[3](#_ENREF_3)] cited by [[4-7](#_ENREF_4)] and Cates 1991 [[15](#_ENREF_15)] cited by [[7](#_ENREF_7)] |
|  | Harrison 1985 [[19](#_ENREF_19)] | Design: Cross-sectional study of 162 female students attending a primary care facility  Limitations: Sample was 5% of new attenders at clinic during study period; data on presence or absence of symptoms not specifically collected  Main findings: 8.0% (13/162) of the sample had positive chlamydia cultures from endocervical swabs; 28.6% (4/14) with a mucopurulent discharge were infected; 33.3% (2/6) with heavy cervical discharge were infected; of the chlamydia positive women, 12.9% (12/93) had cervical erythema, 17.5% (11/63) had cervical ectopy and 25.0% (7/28) had a friable cervix | Stamm 1990 cited by Marrazzo 1997 [[20](#_ENREF_20)] cited by  Kretzschmar 2001 [[3](#_ENREF_3)] cited by [[4-7](#_ENREF_4)] |
|  | Rahm 1986 [[21](#_ENREF_21)] | Article in Swedish | Genc 1993 [[2](#_ENREF_2)] cited by Kretzschmar 2001 [[3](#_ENREF_3)] cited by [[4-7](#_ENREF_4)] |
|  | Stamm 1986 [[22](#_ENREF_22)] | Design: Prospective cohort of 10 men with asymptomatic urethral infection Limitations: Study design is not suitable for determining the proportion of infections that are asymptomatic  Main Findings: "up to one third of these infections detected in prevalence studies have been asymptomatic”, cite Stamm 1984 [[18](#_ENREF_18)] and Podgore 1982 [[8](#_ENREF_8)] | [[7](#_ENREF_7)] and Cates 1991 [[15](#_ENREF_15)] cited by [[7](#_ENREF_7)] |
|  | Karam 1986 [[23](#_ENREF_23)] | Design: Cohort of 85 asymptomatic heterosexual men attending Emergency Department  Limitations: Study design is not suitable for determining the proportion of infections that are asymptomatic  Main findings: 10.6% (11/85) of men had positive chlamydia culture; 57.1% (4/7) of men with a positive culture who also had a MicroTrak test were chlamydia positive on the MicroTrak | [[24](#_ENREF_24)] cited by [[7](#_ENREF_7), [9](#_ENREF_9)]and Kretzschmar 2001[[3](#_ENREF_3)] cited by [[4-7](#_ENREF_4)] |
|  | Edgardh 1987 [[25](#_ENREF_25)] | Article in Swedish | Genc 1993 [[2](#_ENREF_2)] cited by Kretzschmar 2001 [[3](#_ENREF_3)] cited by [[4-7](#_ENREF_4)] |
|  | Hossain 1989 [[26](#_ENREF_26)] | Design: Review  Limitations: Not a systematic review  Main Findings: 50% of uncomplicated infections in women are subclinical, cite Maurice 1983 [[12](#_ENREF_12)]; approximately 20-25% of men have neither signs nor symptoms | [[13](#_ENREF_13)] |
|  | Randolph 1990 [[17](#_ENREF_17)] | Design: Economic analysis of chlamydia screening in adolescent males  Limitations: Not an empirical study of proportion asymptomatic  Main findings: 30% of infected adolescent males are asymptomatic, cite Stamm 1984 [[18](#_ENREF_18)] and Adger 1984 [[27](#_ENREF_27)] | Genc 1993 [[2](#_ENREF_2)] cited by Kretzschmar 2001 [[3](#_ENREF_3)] cited by [[4-7](#_ENREF_4)] |
|  | Stamm 1990 [[28](#_ENREF_28)] | Design: Review  Limitations: Unable to find description of proportion of infections that are asymptomatic in men  Main findings: “at least a third” of women with chlamydia isolated from the cervix have “local signs of infection”, cite Harrison 1985 [[19](#_ENREF_19)] | Marrazzo 1997 [[20](#_ENREF_20)] cited by  Kretzschmar 2001 [[3](#_ENREF_3)] cited by [[4-7](#_ENREF_4)] |
|  | Zimmermann 1990 [[29](#_ENREF_29)] | Design: Prospective cohort of 3078 patients presenting for genital examination  Limitations: Potential selection bias (half of clients were not tested for chlamydia); adjusted case detection rates for sensitivity and specificity to overcome the low sensitivity of diagnostic test  Main findings: Estimate that if culture had been used for diagnosis, nearly 50% of infections in men and 80% of infections in women would have been asymptomatic. 20% of women in the study “presented spontaneously for examination”. | Hillis 1995 [[24](#_ENREF_24)] cited by [[7](#_ENREF_7), [9](#_ENREF_9)] and Kretzschmar 2001 [[3](#_ENREF_3)] cited by [[4-7](#_ENREF_4)] |
|  | Cates 1991 [[15](#_ENREF_15)] | Design: Review  Limitations: Not a systematic review  Main findings: Up to 25% of men (cite Stamm 1984 [[18](#_ENREF_18)] and Stamm 1986 [[22](#_ENREF_22)]) and 75% of women (cite Schacter 1983 [[14](#_ENREF_14)]) are asymptomatic | [[7](#_ENREF_7)] |
|  | Genc 1993 [[2](#_ENREF_2)] | Design: Cost-effectiveness analysis of screening in adolescent men  Limitations: Not an empirical study of proportion asymptomatic  Main findings: Assume “40-60% of infected men would seek medical care for symptomatic urethritis and 1-3% would seek medical care for epididymitis”, cite Randolph 1990 [[17](#_ENREF_17)] and Kaufman 1974 [[1](#_ENREF_1)]; assume “20-50% of infected women would seek medical care for symptomatic cervicitis and 8-15% would seek medical care for acute PID”, cite Thelin 1982 [[10](#_ENREF_10)], Rahm 1986 [[21](#_ENREF_21)], Edgardh 1987 [[25](#_ENREF_25)] and Westrom 1982 [[11](#_ENREF_11)] | Kretzschmar 2001 [[3](#_ENREF_3)] cited by [[4-7](#_ENREF_4)] |
|  | Stamm 1993 [[30](#_ENREF_30)] | Design: Review/evidence summary  Limitations: Not a systematic review  Main findings: No figures presented | Marrazzo 1997 [[20](#_ENREF_20)] cited by  Kretzschmar 2001 [[3](#_ENREF_3)] cited by [[4-7](#_ENREF_4)] |
|  | Hillis 1995 [[24](#_ENREF_24)] | Design: Review  Limitations: Not a systematic review  Main Findings: “approximately 70% of women with endocervical infections and up to 50% of men with urethral infections are asymptomatic”, cites Karam 1986 [[23](#_ENREF_23)], Podgore 1982 [[8](#_ENREF_8)], Stamm 1990 [[28](#_ENREF_28)] and Zimmerman 1990 [[29](#_ENREF_29)] | [[7](#_ENREF_7), [9](#_ENREF_9)] and Kretzschmar 2001 [[3](#_ENREF_3)] cited by [[4-7](#_ENREF_4)] |
|  | Schachter 1997 [[31](#_ENREF_31)] | Design: Clinical case study and perspective article  Limitations: No reference provided for the presented figures  Main findings: “most infected women – and about half of infected men – are initially asymptomatic or have only mild symptoms and, so, are not treated”; “approximately 70% of women found to have chlamydia infection during screening have neither signs nor symptoms of the disease.” | [[7](#_ENREF_7)] and Kretzschmar 2001 [[3](#_ENREF_3)] cited by [[4-7](#_ENREF_4)] |
|  | Marrazzo 1997 [[20](#_ENREF_20)] | Design: Cross-sectional study and cost-effectiveness analysis of screening criteria  Limitations: Not an empirical study of proportion asymptomatic  Main findings: “Only 10% to 30% of reported chlamydial infections in women are symptomatic”, cite Stamm 1990 [[28](#_ENREF_28)] and Stamm 1993 [[30](#_ENREF_30)] | Kretzschmar 2001 [[3](#_ENREF_3)] cited by [[4-7](#_ENREF_4)] |
|  | Van der Laar 1997 [[32](#_ENREF_32)] | Design: Cross sectional study of partner notification in 355 people at an STI clinic  Limitations: Data for chlamydia and gonorrhoea are not presented separately  Main findings: 60.5% (23/38) of asymptomatic female partners were infected [assume with either chlamydia or gonorrhoea] and 71.4% (10/14) of asymptomatic male partners were infected | Kretzschmar 2001 [[3](#_ENREF_3)] cited by [[4-7](#_ENREF_4)] |
|  | Van Duynhoven 1998 [[33](#_ENREF_33)] | Design: Cross sectional study of 495 sexual health clinic attendees and their reported contacts (52 female and 48 heterosexual male)  Limitations: Data for chlamydia and gonorrhoea are not presented separately  Main findings: 58.3% (14/24) of female partners and 72.2% (13/18) of male partners testing positive for chlamydia or gonorrhoea were asymptomatic | Kretzschmar 2001 [[3](#_ENREF_3)] cited by [[4-7](#_ENREF_4)] |
|  | Kretzschmar 2001 [[3](#_ENREF_3)] | Design: Mathematical modelling study of the impact of chlamydia screening that identified parameter values through literature search  Limitations: Not an empirical study of proportion asymptomatic  Main findings: 50% of infections in men and 70% in women are asymptomatic, cite Hillis 1995 [[24](#_ENREF_24)], Schacter 1997 [[31](#_ENREF_31)], Genc 1993 [[2](#_ENREF_2)], Marrazzo 1997 [[20](#_ENREF_20)], van der Laar 1997 [[32](#_ENREF_32)], van Duynhoven 1998 [[33](#_ENREF_33)] | [[4-7](#_ENREF_4)] |
|  | Turner 2002 [[34](#_ENREF_34)] | Design: Cross-sectional behavioural survey of Baltimore households, collected urine from 579 adults aged 18-35 years  Limitations: Excluded people treated for chlamydia or gonorrhoea in previous 6 months  Main findings: 2% of currently infected respondents (with either chlamydia or gonorrhoea) reported dysuria and 4.7% reported discharge within the past 6 months | [[35](#_ENREF_35)] |
|  | Golden 2003 [[36](#_ENREF_36)] | Design: Interviews with clinic staff to determine the proportion of cases of HIV and STI where partner notification was conducted  Limitations: Study design is not suitable for determining the proportion of infections that are asymptomatic  Main Findings: Partner notification occurred for 14% of patients with chlamydia | [[35](#_ENREF_35)] |
|  | Turner 2006 [[37](#_ENREF_37)] | Design: Mathematical modelling study that obtained parameter through model fitting to observed prevalence of chlamydia and the proportion of individuals who report ever being diagnosed with Chlamydia in the UK using NATSAL data [[38](#_ENREF_38)]and a systematic review of prevalence [[39](#_ENREF_39)]  Limitations: Accuracy of fit parameters is dependent on representativeness of data used to fit the model and assumptions made in the model  Main findings: 95.5% of infections in women and 100% of infections in men were estimated to be asymptomatic | [[40](#_ENREF_40), [41](#_ENREF_41)] |
|  | Tao 2007 [[42](#_ENREF_42)] | Design: National Survey for Family Growth in U.S., data from 2563 young women used to estimate chlamydia screening rates  Limitations: Survey question related to all STIs, not specifically chlamydia; study design is not suitable for determining the proportion of infections that are asymptomatic  Main findings: 42% of sexually active women reported being tested for STIs | [[35](#_ENREF_35)] |
| Duration of infection | Holmes 1974 [[43](#_ENREF_43)] | Design: Review  Limitations: Not a systematic review  Main findings: During a six month follow up study of asymptomatic men, most remained carriers at end of study, cite presentation by Handsfield et al 1972 [[44](#_ENREF_44)] | Wiesner et al 1980 [[45](#_ENREF_45)] cited by van der Laar 1993 [[46](#_ENREF_46)] cited by Kretzschmar 2001 [[3](#_ENREF_3)] cited by [[4-7](#_ENREF_4), [9](#_ENREF_9)] |
|  | Wiesner et al 1980 [[45](#_ENREF_45)] | Design: Review  Limitations: References not cited for key parameters  Main findings: Women “develop…symptoms…within 3-45 days of infection”; in men the “perceived incubation period [is] 2 to 30 days”; untreated urethritis “usually resolves spontaneously within 6 months”, cite Holmes 1974 [[43](#_ENREF_43)] | van der Laar 1993 [[46](#_ENREF_46)] cited by Kretzschmar 2001 [[3](#_ENREF_3)] cited by [[4-7](#_ENREF_4), [9](#_ENREF_9)] |
|  | Rahm 1986 [[47](#_ENREF_47)] | Design: Prospective cohort of 109 asymptomatic chlamydia positive women aged 15-20 years, untreated until 3 months after diagnosis  Limitations: culture used for diagnosis; follow up limited to 3 months for majority of cohort  Main findings: 79.8% (87/109) remained asymptomatic at 3 months; 80.5% (70/87) of these women were culture positive at 3 months; 64.2% (70/109) were asymptomatic culture positive at 3 months; 18 (16.5%) women developed symptoms by 3 months of whom 9 (56.3%) remained culture positive; possibly 3 (2.8%) lost to follow up | Buhaug 1989 [[48](#_ENREF_48)] cited by Kretzschmar 2001 [[3](#_ENREF_3)] cited by [[4-7](#_ENREF_4), [9](#_ENREF_9)] and Buhaug 1990 cited by [[9](#_ENREF_9)] |
|  | Buhaug 1989 [[48](#_ENREF_48)] | Design: Cost-effectiveness study of non-routine testing for chlamydia  Limitations: Not an empirical study of duration of infection  Main findings: use an average duration of infection of 52 weeks (95% CI 35 – 88 weeks), based on Rahm 1986 [[47](#_ENREF_47)] | Kretzschmar 2001 [[3](#_ENREF_3)] cited by [[4-7](#_ENREF_4), [9](#_ENREF_9)] |
|  | Buhaug 1990 [[49](#_ENREF_49)] | Design: Cost-effectiveness study of screening asymptomatic people for chlamydia in general practice  Limitations: Not an empirical study of duration of infection  Main findings: use an average duration of infection of 52 weeks, based on Rahm 1986 [[47](#_ENREF_47)] | [[9](#_ENREF_9)] |
|  | van der Laar 1993 [[46](#_ENREF_46)] | Design: Technical report with literature review  Limitations: Not an empirical study of duration of infection  Main findings: Present data on gonorrhoea - duration of symptomatic infection in women and infection (symptomatic and asymptomatic) in men | Kretzschmar 2001 [[3](#_ENREF_3)] cited by [[4-7](#_ENREF_4), [9](#_ENREF_9)] |
|  | Golden 2000 [[50](#_ENREF_50)] | Design: Review  Limitations to included studies: Unknown date of infection; reinfection not considered; length time bias of screening  Main findings: Not possible to reliably estimate duration of infection; most infections in women persist for more than 60 days; age and symptoms may be associated with higher clearance rates; duration of infection may be shorter in men but evidence inconclusive | Turner 2006 [[37](#_ENREF_37)] cited by [[40](#_ENREF_40)], cited by [[41](#_ENREF_41)] |
|  | Kretzschmar 2001 [[3](#_ENREF_3)] | Design: As above  Limitations: Not an empirical study of duration of infection  Main findings: Duration of asymptomatic infection is 200 days in men and 370 days in women; duration of symptomatic infection is 33 days in men and 40 days in women, cite Buhaug 1989 [[48](#_ENREF_48)] and Rahm 1986 [[47](#_ENREF_47)] | [[4-7](#_ENREF_4)] |
|  | Korenromp 2002 [[51](#_ENREF_51)] | Design: Secondary analysis of data from cross-sectional clinical studies in Uganda  Limitations: Duration of infection is not a primary outcome; cross sectional study design; low sensitivity of culture (although results adjusted); generalizability (very low rate of treatment in study populations)  Main findings: Non-weighted pooled (symptomatic and asymptomatic infection) estimates suggest a mean duration of infection of 132 days for men and 499 days for women | Turner 2006 [[37](#_ENREF_37)] cited by [[40](#_ENREF_40)], cited by [[41](#_ENREF_41)] |
|  | Turner 2006 [[37](#_ENREF_37)] | Design and limitations: As above  Main findings: Duration of infection 30 days in treatment seeking individuals and 180 days in non-treatment seeking individuals. Figures are an assumption based on Golden 2000[[50](#_ENREF_50)] and Korenromp 2002 [[51](#_ENREF_51)] | [[40](#_ENREF_40)], cited by [[41](#_ENREF_41)] |
|  | Geisler 2010 [[52](#_ENREF_52)] | Design: Review  Limitations to included studies: unknown date of infection; unable to distinguish re-infection from persistent infection; generalizability (study populations often have high chlamydia prevalence)  Main findings: Clearance of infection increases with time and approximately half spontaneously resolved around 1 year after the initial chlamydia test; no clear evidence about whether men or women clear the infection faster | [[35](#_ENREF_35)] |
| Per act probability of transmission | Katz 1992 [[53](#_ENREF_53)] | Design: Mathematical estimation of the per act transmission probability using data from a contact tracing programme  Limitations: Does not consider reinfection within partnerships and is determined during the partnership; dependant on number of sex acts within partnership  Main findings: Per partnership transmission probability of 0.395 from men to women and 0.323 from women to men | Kretzschmar 2001 [[3](#_ENREF_3)] cited by [[4-7](#_ENREF_4)] |
|  | Quinn 1996 [[54](#_ENREF_54)] | Design: Study of concordance in sexual partnerships attending STI clinics in US where the presenting partner tested positive for chlamydia using PCR  Limitations: Does not take into account the probability of two infected people forming a partnership; measured during the partnership rather than at the end  Main findings: 68% of male partners (53/78) of women who tested positive for chlamydia also tested positive and 70% of female partners (53/76) of men who tested positive for chlamydia also tested positive | [[9](#_ENREF_9)] and Kretzschmar 2001 [[3](#_ENREF_3)] cited by [[4-7](#_ENREF_4)] |
|  | Kretzschmar 2001 [[3](#_ENREF_3)] | Design: As above  Limitations: Not an empirical study of transmission probability  Main findings: Use a per contact transmission probability of 0.11, cite Quinn 1996 [[54](#_ENREF_54)] and Katz 1992 [[53](#_ENREF_53)] | [[4-7](#_ENREF_4)] |
|  | Turner 2006 [[37](#_ENREF_37)] | Design and limitations: As above  Main findings: Per act transmission probability of 0.0375 | [[40](#_ENREF_40)] cited by [[41](#_ENREF_41)] |

**References**

1. Kaufman, R.E. and P.J. Wiesner, *Nonspecific Urethritis.* New England Journal of Medicine, 1974. **291**(22): p. 1175-1177.

2. Genc, M., L. Ruusuvaara, and P.A. Mardh, *AN ECONOMIC-EVALUATION OF SCREENING FOR CHLAMYDIA-TRACHOMATIS IN ADOLESCENT MALES.* Jama-Journal of the American Medical Association, 1993. **270**(17): p. 2057-2064.

3. Kretzschmar, M., et al., *Comparative model-based analysis of screening programs for Chlamydia trachomatis infections.* American Journal of Epidemiology, 2001. **153**(1): p. 90-101.

4. Andersen, B., et al., *Prediction of costs, effectiveness, and disease control of a population-based program using home sampling for diagnosis of urogenital Chlamydia trachomatis infections.* Sexually Transmitted Diseases, 2006. **33**(7): p. 407-415.

5. Low, N., et al., *Epidemiological, social, diagnostic and economic evaluation of population screening for genital chlamydial infection.* Health Technol Assess, 2007. **11**(8): p. iii-iv, ix-xii, 1-165.

6. Roberts, T.E., et al., *Cost effectiveness of home based population screening for Chlamydia trachomatis in the UK: economic evaluation of chlamydia screening studies (ClaSS) project.* British Medical Journal, 2007. **335**(7614): p. 291-294A.

7. Welte, R., et al., *Costs and effects of chlamydial screening: dynamic versus static modeling.* Sex Transm Dis, 2005. **32**(8): p. 474-83.

8. Podgore, J.K., K.K. Holmes, and E.R. Alexander, *Asymptomatic urethral infections due to Chlamydia trachomatis in male U.S. military personnel.* J Infect Dis, 1982. **146**(6): p. 828.

9. de Vries, R., et al., *Cost-utility of repeated screening for chlamydia trachomatis.* Value in Health, 2008. **11**(2): p. 272-274.

10. Thelin I., M.P.-A., *Contact tracing in genital chlamdial infection*, in *Chlamydia trachomatis in Genital and Related Infections*, M.B. Mardh P-A., Paavonen J., Editor. 1982, Almqvist & Wiksell International: Stockholm, Sweden. p. 163-166.

11. Westrom, L., et al., *Chlamydial and gonococcal infections in a defined population of women.* Scand J Infect Dis Suppl, 1982. **32**: p. 157-62.

12. Maurice, J., *Ubiquitous parasites: Chlamydial infections now commonest sex-transmitted disease.* Int Health Mag, 1983. **3**: p. 19.

13. Townshend, J.R.P. and H.S. Turner, *Analysing the effectiveness of Chlamydia screening.* Journal of the Operational Research Society, 2000. **51**(7): p. 812-824.

14. Schachter J, S.E., Moncada J., *Screening for chlamydial infection is women attending family planning clinics.* West J Med, 1983. **138**: p. 375-9.

15. Cates, W., Jr. and J.N. Wasserheit, *Genital chlamydial infections: epidemiology and reproductive sequelae.* Am J Obstet Gynecol, 1991. **164**(6 Pt 2): p. 1771-81.

16. Adger, H., et al., *Screening for Chlamydia trachomatis and Neisseria gonorrhoeae in adolescent males: value of first-catch urine examination.* Lancet, 1984. **2**(8409): p. 944-5.

17. Randolph, A.G. and A.E. Washington, *Screening for Chlamydia trachomatis in adolescent males: a cost-based decision analysis.* Am J Public Health, 1990. **80**(5): p. 545-50.

18. Stamm, W.E., et al., *Chlamydia trachomatis urethral infections in men. Prevalence, risk factors, and clinical manifestations.* Ann Intern Med, 1984. **100**(1): p. 47-51.

19. Harrison, H.R., et al., *Cervical Chlamydia trachomatis infection in university women: relationship to history, contraception, ectopy, and cervicitis.* Am J Obstet Gynecol, 1985. **153**(3): p. 244-51.

20. Marrazzo, J.M., et al., *Performance and cost-effectiveness of selective screening criteria for Chlamydia trachomatis infection in women - Implications for a national chlamydia control strategy.* Sexually Transmitted Diseases, 1997. **24**(3): p. 131-141.

21. Rahm V., G.A., Rosen G., *Chlamydial infection is common among teenage girls who seek contraceptive advice.* Lakartidningen, 1986. **83**: p. 615-616.

22. Stamm, W.E. and B. Cole, *Asymptomatic Chlamydia trachomatis urethritis in men.* Sex Transm Dis, 1986. **13**(3): p. 163-5.

23. Karam, G.H., et al., *Asymptomatic Chlamydia Trachomatis Infections among Sexually Active Men.* The Journal of Infectious Diseases, 1986. **154**(5): p. 900-903.

24. Hillis, S., et al., *NEW OPPORTUNITIES FOR CHLAMYDIA PREVENTION - APPLICATIONS OF SCIENCE TO PUBLIC-HEALTH PRACTICE.* Sexually Transmitted Diseases, 1995. **22**(3): p. 197-202.

25. Edgardh K., H.U., Bygdeman S., Sandstrom E., Backman M. , *Asymptomatic teenage girls should be tested for chlamydial infection* Lakartidningen, 1987. **84**: p. 1854-1847.

26. Hossain, A., *CHLAMYDIA-TRACHOMATIS INFECTIONS.* International Journal of Gynecology & Obstetrics, 1989. **29**(2): p. 107-115.

27. Adger, H., et al., *SCREENING FOR CHLAMYDIA TRACHOMATIS AND NEISSERIA GONORRHOEAE IN ADOLESCENT MALES: VALUE OF FIRST-CATCH URINE EXAMINATION.* The Lancet, 1984. **324**(8409): p. 944-945.

28. Stamm, W.E., Holmes, K. K., *Chlamydia trachomatis infections of the adult*, in *Sexually Transmitted Diseases*, K.K. Holmes, Editor. 1990, McGraw-Hill: USA. p. 181-194.

29. Zimmerman, H.L., et al., *Epidemiologic differences between chlamydia and gonorrhea.* Am J Public Health, 1990. **80**(11): p. 1338-42.

30. Stamm, W.E., *Toward control of sexually transmitted chlamydial infections.* Ann Intern Med, 1993. **119**(5): p. 432-4.

31. Schachter J, M., Sweet RL, et al. , *Control of Chlamydia: a role for every clinician who sees sexually active patients.* J Clin Out Manag, 1997. **4**: p. 60-73.

32. van de Laar, M.J.W., F. Termorshuizen, and A. VandenHoek, *Partner referral by patients with gonorrhea and chlamydial infection - Case-finding observations.* Sexually Transmitted Diseases, 1997. **24**(6): p. 334-342.

33. van Duynhoven, Y.T.H.P., et al., *Patient referral outcome in gonorrhoea and chlamydial infections.* Sexually Transmitted Infections, 1998. **74**(5): p. 323-330.

34. Turner, C.F., et al., *Untreated gonococcal and chlamydial infection in a probability sample of adults.* JAMA, 2002. **287**(6): p. 726-33.

35. Tuite, A.R., et al., *Estimation of the Burden of Disease and Costs of Genital Chlamydia trachomatis Infection in Canada.* Sex Transm Dis, 2012. **39**(4): p. 260-7.

36. Golden, M.R., et al., *Partner notification for HIV and STD in the United States: low coverage for gonorrhea, chlamydial infection, and HIV.* Sex Transm Dis, 2003. **30**(6): p. 490-6.

37. Turner, K.M., et al., *Developing a realistic sexual network model of chlamydia transmission in Britain.* Theor Biol Med Model, 2006. **3**: p. 3.

38. Johnson, A.M., et al., *Sexual behaviour in Britain: partnerships, practices, and HIV risk behaviours.* Lancet, 2001. **358**(9296): p. 1835-42.

39. Adams, E.J., et al., *Chlamydia trachomatis in the United Kingdom: a systematic review and analysis of prevalence studies.* Sexually Transmitted Infections, 2004. **80**(5): p. 354-362.

40. Adams, E.J., K.M. Turner, and W.J. Edmunds, *The cost effectiveness of opportunistic chlamydia screening in England.* Sex Transm Infect, 2007. **83**(4): p. 267-74; discussion 274-5.

41. Gillespie, P., et al., *The cost and cost-effectiveness of opportunistic screening for Chlamydia trachomatis in Ireland.* Sex Transm Infect, 2012. **88**(3): p. 222-8.

42. Tao, G., L.H. Tian, and T.A. Peterman, *Estimating Chlamydia screening rates by using reported sexually transmitted disease tests for sexually active women aged 16 to 25 years in the United States.* Sex Transm Dis, 2007. **34**(3): p. 180-2.

43. Holmes, K.K., *Gonococcal infection. Clinical, epidemiologic and laboratory perspectives.* Adv Intern Med, 1974. **19**: p. 259-85.

44. Handsfield, H.H., Lipman, T., Harnisch, J. P. et al, *Diagnosis, prevalence, and natural history of asymptomatic gonorrhea in the male* 1972: Presented at the Twelfth Interscience Conference on Antimicrobial Agents and Chemotherapy, Atlantic City.

45. Wiesner, P.J. and S.E. Thompson, 3rd, *Gonococcal diseases.* Dis Mon, 1980. **26**(5): p. 1-44.

46. van de Laar, M.J.W., *Seksueel Overdraagbare Aandoeningen In Nederland*, 1993, Rijksinstituut Voor Volksgezondheid En Milieuhygiene Bilthoven.

47. Rahm, V.A., et al., *ASYMPTOMATIC CARRIAGE OF CHLAMYDIA-TRACHOMATIS - A STUDY OF 109 TEENAGE GIRLS.* European Journal of Sexually Transmitted Diseases, 1986. **3**(2): p. 91-94.

48. Buhaug, H., et al., *Cost-Effectiveness of Testing for Chlamydial Infections in Asymptomatic Women.* Medical Care, 1989. **27**(8): p. 833-841.

49. Buhaug, H., et al., *Should asymptomatic patients be tested for Chlamydia trachomatis in general practice?* Br J Gen Pract, 1990. **40**(333): p. 142-5.

50. Golden, M.R., et al., *Duration of untreated genital infections with chlamydia trachomatis: a review of the literature.* Sex Transm Dis, 2000. **27**(6): p. 329-37.

51. Korenromp, E.L., et al., *What proportion of episodes of gonorrhoea and chlamydia becomes symptomatic?* Int J STD AIDS, 2002. **13**(2): p. 91-101.

52. Geisler, W.M., *Duration of untreated, uncomplicated Chlamydia trachomatis genital infection and factors associated with chlamydia resolution: a review of human studies.* J Infect Dis, 2010. **201 Suppl 2**: p. S104-13.

53. Katz, B., *Estimating transmission probabilities for chlamydial infection.* Statist Med, 1992. **11**: p. 565-577.

54. Quinn, T.C., et al., *Epidemiologic and microbiologic correlates of Chlamydia trachomatis infection in sexual partnerships.* JAMA, 1996. **276**(21): p. 1737-42.
